# Relaxation dynamics in the strong chalcogenide glass-former of Ge_22_Se_78_

**DOI:** 10.1038/srep40547

**Published:** 2017-01-17

**Authors:** Pengfei Li, Yaqi Zhang, Zeming Chen, Peng Gao, Tao Wu, Li-Min Wang

**Affiliations:** 1 State Key Lab of Metastable Materials Science and Technology, and College of Materials Science and Engineering, Yanshan University, Qinhuangdao, Hebei, 066004, China

## Abstract

The enthalpy relaxation is performed in the glassy Ge_22_Se_78_ to understand the dynamic behaviors. The structure of the glass is examined by X-ray diffraction and Raman spectra. The dynamic parameters such as the fragility, stretching exponent and non-linear factor are determined. A low fragility of *m* = 27 is exhibited for the chalcogenide, however, the stretching exponent is found not to have a larger value. The enthalpy relaxation spectra are constructed for various glass formers, and a relationship between the fragility and the symmetry of the spectra is demonstrated. The dynamic results are used to evaluate the structure of the Ge_22_Se_78_ glass.

Ge_*x*_Se_1−*x*_ chalcogenide glasses exhibit superior optical properties in the infrared region, and have been considerably applied in the industries such as solar cells and infrared optical fibers^1^,^2^. Due to the non-equilibrium nature of glassy materials and the tendency moving towards equilibrium states or stable states to release excess energy, the structure and properties might be subjected to variation at a specific temperature, which is initiated by the structural relaxation^3^. Therefore, the stability of glassy materials is directly related to the structural relaxation dynamics, which is critical to understand the property changes in such materials. The relaxation dynamics of the Se-rich Ge_*x*_Se_1−*x*_ glasses have been explored for decades^4^,^5^,^6^, however, a complete interpretation is still needed. In addition, the structure in Ge_*x*_Se_1−*x*_ glasses has been a subject of discussion concerning the connection patterns of the basic structural motifs of GeSe_4_ tetrahedra and (Se)_n_ units^7^,^8^,^9^. Besides the direct structure analyses method such as the diffraction methods^10^ and NMR spectroscopy^11^, dynamic studies have proven to be capable of offering insights into the structure of glasses^12^,^13^.

Generally, the structural relaxation in glassy materials is largely governed by three dynamic aspects covering non-Arrhenius, non-exponential and non-linear behaviors^12^. The fragility *m*-index reflects how rapidly liquid viscosity or structural relaxation time changes at the glass transition temperature *T*_*g*_, and is quantified by *d*log_10_(*x*)/*d*(*T*_*g*_*/T*) at *T* = *T*_*g*_ in Angell plot, where *x* denotes the viscosity or the relaxation time^14^. The stretching exponent (or non-exponential factor) *β*_*KWW*_ quantifies the degree of the non-exponential dynamics and is usually involved in the isothermal Kohlrausch-William-Watts (KWW) function in the time domain, 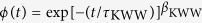
15, where *τ*_*KWW*_ is the structural relaxation time, and *β*_*KWW*_ is the stretching exponent with 0 < *β*_*KWW*_ ≤ 1^16^,^17^,^18^. The non-linear factor defines how much the structural relaxation dynamics in glassy states depends on the thermal history.

Considerable efforts have been made to explore the dynamics in the Ge_*x*_Se_1−*x*_ melts and glasses, however, the reported results are not consistent. Based on the viscosity data of the Ge_*x*_Se_100−*x*_ melts, the fragility in the compositions of 10 < *x* < 25 is calculated to range from 22.5 to 32^4^,^5^,^19^. The enthalpy relaxation studies of Ge_8_Se_92_ and Ge_12_Se_88_ reported *m* = 58–59^20^. For the Ge_*x*_Se_100−*x*_ alloys with 10 < *x* < 33, the fragility *m* index was reported to be 14.8–29 using the modulated differential scanning calorimetry (mDSC)^6^. Recent studies of the enthalpy relaxation in Ge_*x*_Se_1−*x*_ (*x* ≤ 15) presented a continuous decrease in fragility from 68 to 28 measured with a constant ratio between the cooling and subsequent heating rates^21^. Similarly, the *β*_*KWW*_ values (or the non-linear factor *x*) determined in the enthalpy relaxation measurements are not consistent. It is also found that the values of *β*_*KWW*_ and *x* determined by the enthalpy relaxation are notably higher than those determined by the volume relaxation^22^.

Given accurate dynamic parameters are crucial to understand the relaxation behaviors and the structure in glassy materials^12^,^15^, here, the structural relaxation dynamics of the Ge_22_Se_78_ chalcogenide glass is studied, referring to earlier studies in Ge_*x*_Se_1−*x*_ where a minimum fragility is identified to fall in the compositions of 19 < *x* < 26, preferentially in the range of 21.5 < *x* < 23^4^,^5^,^6^. The dynamic parameters *m, β*_*KWW*_ and *x* are determined using the enthalpy relaxation, because the method has proven to be capable of producing quite comparable results with the viscosity or dielectric-relaxation measurements^23^,^24^. The fragility of the Ge_22_Se_78_ glass is determined to be low with *m* = 27. Surprisingly, a low stretching exponent is also revealed. The dynamic results are used to understand the structure of the Ge_22_Se_78_ glass.

## Results

**Figure 1:**
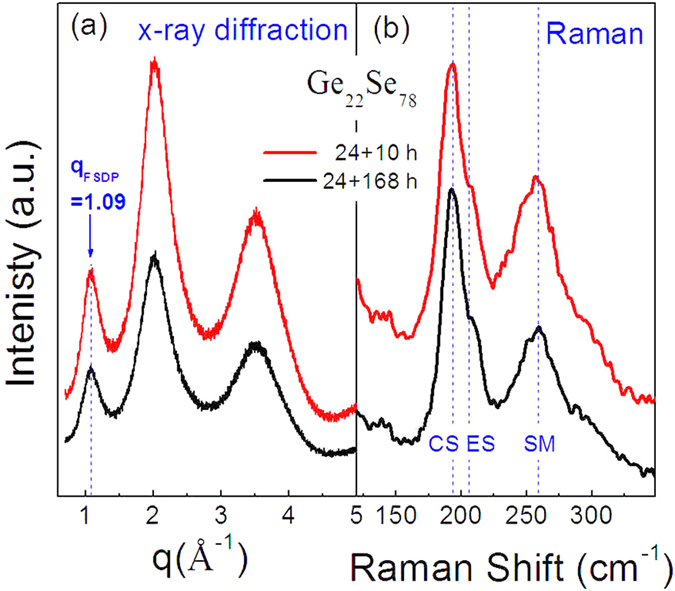
(**a**) XRD patterns of Ge_22_Se_78_ with different synthesis time before quenching (**b**) Raman spectra of Ge_22_Se_78_ at room temperature. (CS) in the Raman spectrum denotes the corner-shared GeSe_4_ tetrahedra, (ES) denotes the vibration of Se atoms in edge-shared GeSe_4_ tetrahedra, and (SM) is Se-Se stretching modes in (Se)_n_ units.

Figure 1(a) and (b) present the XRD patterns and Raman spectra of the glassy Ge_22_Se_78_ samples synthesized for 24 + 10 hours (details are presented in the Sample preparation section) and 24 + 168 hours. The nature of completely amorphous states is identified for the two samples by the XRD patterns using the wave vector gauged by *q* = 4*π* sin*θ/λ*. The intermediate-range order is unambiguously observed by the featured first-sharp-diffraction-peak (FSDP) at *q* = 1.09 Å^−1^, given that the FSDPs in many non-metallic network glassy materials are typically located at *q* ~ 1–2 Å^−1 ^25. The Raman spectra displayed in Fig. 1(b) show two main regions: (a) a relatively sharp band with high intensity at ~195 cm^−1^ along with a shoulder located at ~210 cm^−1^ and (b) a broad band centered at 259 cm^−1^ with high-intensity, spanning from 225 to 280 cm^−1^. The 195 cm^−1^ band in region (a) is assigned specifically to the breathing mode of corner-shared (CS) GeSe_4_ tetrahedral units, and the 210 cm^−1^ band is assigned to the vibration of Se atoms involved in edge shared (ES) GeSe_4_ tetrahedra^26^,^27^. The band in region (b) around 259 cm^−1^ is characteristic of pure Se and can be assigned to Se-Se stretching modes (SM) in (Se)_n_ units^28^,^29^. Considering the similarity of Raman spectra between the glasses synthesized for 24 + 10 hours and 24 + 168 hours in the rocking furnace, the homogeneous glasses are expected to be achieved.

**Figure 2:**
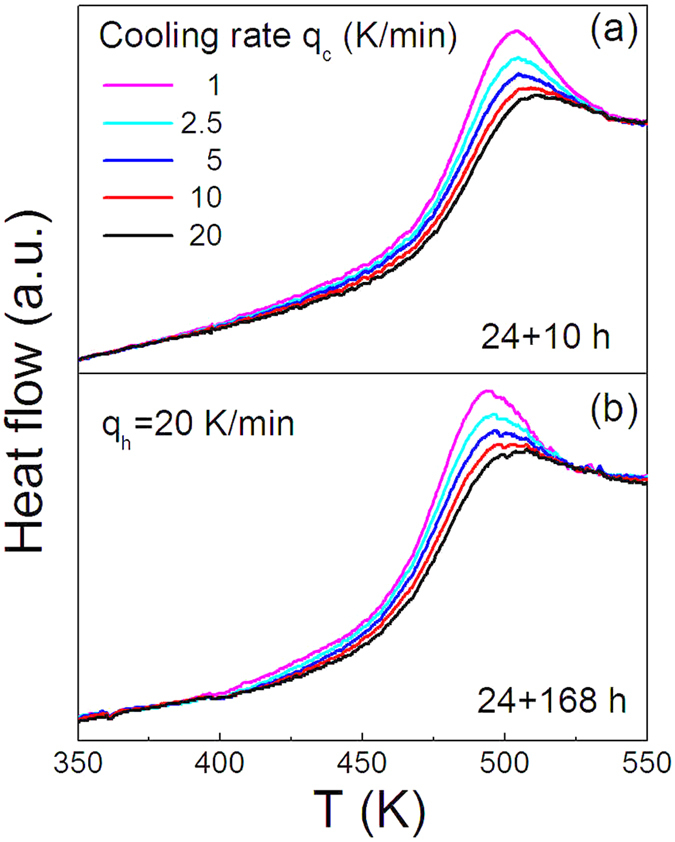
Heating heat flow curves of Ge_22_Se_78_ glasses quenched from various cooling rates, −1, −2.5, −5, −10, and −20 K/min across glass transition (from top). The heating rates are fixed to be 20 K/min. (**a**) The sample with the synthesis time of 24 + 10 hours before quenching into ice water; (**b**) The sample with the synthesis time of 24 + 168 hours.

**Figure 3:**
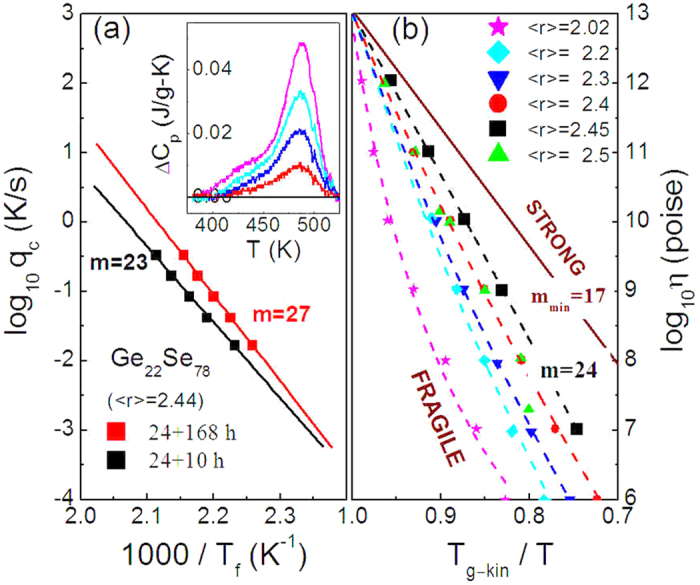
(**a**) Dependence of fictive temperature on cooling rate, which presents fragility *m*-indexes to be 23 and 27 respectively for the Ge_22_Se_78_ synthesized for 24 + 10 and 24 + 168 hours before quenching. The inset of (**a**) shows *C*_*p*_ difference between the glasses quenched from cooling rate of 20 K/min and the glasses quenched at other rates; (**b**) Fragility curves of glass formers from the Ge_*x*_Se_1−*x*_ system and the data were adapted from ref. 4. 〈*r*〉 = 2.5, 2.45, 2.4, 2.3, 2.2 and 2.02 correspond to the composition Ge_0.25_Se_0.75_, Ge_0.225_Se_0.775_, Ge_0.2_Se_0.8_, Ge_0.15_Se_0.85_, Ge_0.1_Se_0.9_ and Ge_0.01_Se_0.99_, respectively.

Figure 2(a) and (b) show the heating heatflow curves around the glass transition for the two glassy Ge_22_Se_78_ samples synthesized for 24 + 10 hours and 24 + 168 hours before quenching. Comparing the curves in the two panels, it is seen that the onset glass transition temperature of the sample synthesized for 24 + 168 hours is a bit lower. The heatflow curves are recorded at a fixed heating rate of 20 K/min after the glasses are quenched at different cooling rates of 20, 10, 5, 2.5 and 1 K/min, from which the fictive temperatures are determined. The fictive temperature, *T*_*f*_, firstly proposed by Tool^30^, and used to serve as an indicator of the states of glasses^23^,^31^, are subsequently determined. The *T*_*f*_ of glasses can be calculated from the enthalpy-matching method for the heating curves using Moynihan’s construction, following the relation, 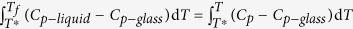, where T* is an arbitrary temperature above *T*_*g*_, *C*_*p-liquid*_ and *C*_*p-glass*_ denote the heat capacity of liquid and glassy states, respectively. Principally, *T*_*f*_ of each glass quenched at a specific cooling rate can be independently calculated from the heating curve. In order to improve the accuracy, we chose the enthalpy-compensation method, 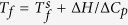, where 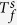 is the fictive temperature of a reference glass quenched at specific cooling/heating rates (i.e., −/+ 20 K/min), Δ*H* is the energy difference between the glass denoted by *T*_*f*_ and the reference glass by 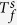, and can be determined from the integral of the *C*_*p*_ difference between the two glasses, Δ*C*_*p*_ is the *C*_*p*_ difference between the liquid and the glass at *T*_*g*_, which is shown in the inset of Fig. 3(a)^23^.

Figure 3(a) shows the dependence of the fictive temperature *T*_*f*_ on the cooling rate *Q* for the two glassy samples subjected to distinct synthesis time in melts. Using the expression of ln *Q* = *A* − *E*_*g*_/*RT*_*f*_, where *R* is the gas constant, *A* a constant, and *E*_*g*_ the apparent glass transition activation energy^23^, the fragility *m*-index is determined to be 23 ± 2 for the glass synthesized for 24 + 10 hours and 27 ± 2 for the 24 + 168 hours in terms of *m* = *E*_*g*_/2.303*RT*_*g*_^32^,^33^. The viscosity of Ge_*x*_Se_1−*x*_ systems are also reproduced in Fig. 3(b) from early references^4^,^5^,^19^ as a function of reciprocal temperature, giving the fragility minimum of *m* = 24 at *x* = 0.225. The fragility determined in the present work are largely comparable with the reported results based on different methods such as viscosity^4^,^5^,^19^ and DSC measurements^6^.

**Figure 4:**
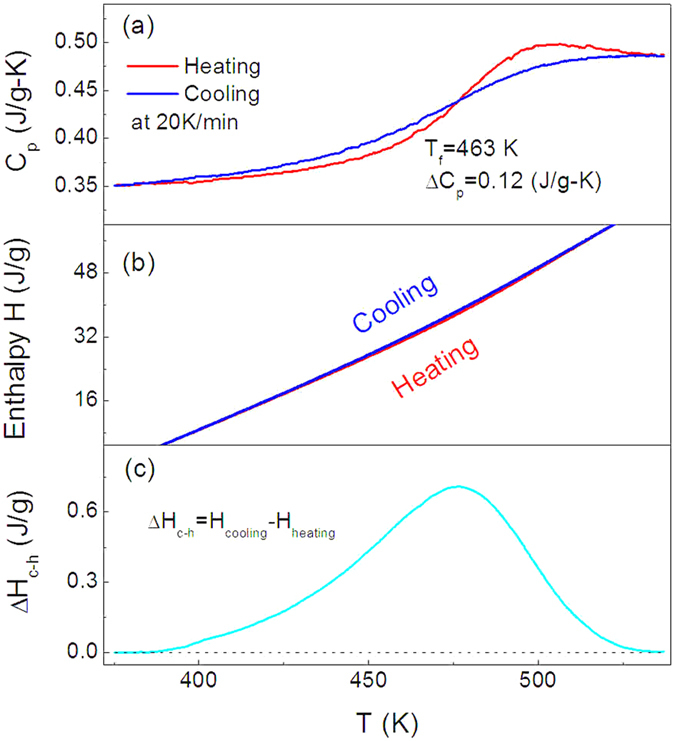
(**a**) Heat capacity curves of Ge_22_Se_78_ synthesized for 24 + 168 hours in the vicinity of glass transition during the cooling and heating measurements at the cooling/heating rates of −/+20 K/min. (**b**) Enthalpy curves in the cooling and reheating cycle. (**c**) Enthalpy difference involved in the cooling and reheating cycle, indicating the enthalpy relaxation and enthalpy recovery in the quenched glasses upon reheating.

The cooling/heating capacity *C*_*p*_ curves of the Ge_22_Se_78_ glass with synthesis time of 24 + 168 hours are shown in Fig. 4(a) using the rates of −/+20 K/min, and the corresponding enthalpies calculated by the integral of the *C*_*p*_ curves, are presented in Fig. 4(b), showing a clear enthalpy hysteresis in the cycle. The hysteresis behaviors involved around glass transition during the cooling and heating cycles have been reported in the enthalpy and optical absorptivity measurements^34^,^35^, and ascribed to the relaxation in glass upon heating (energy release) in glass and subsequent recovery towards equilibrium supercooled liquid at temperature well higher than *T*_*g*_^36^. The enthalpy relaxation spectrum of the Ge_22_Se_78_ glass is shown in Fig. 4(c), giving the maximum relaxation enthalpy, Δ*H*_*R*_ to be 0.708 J/g.

**Figure 5:**
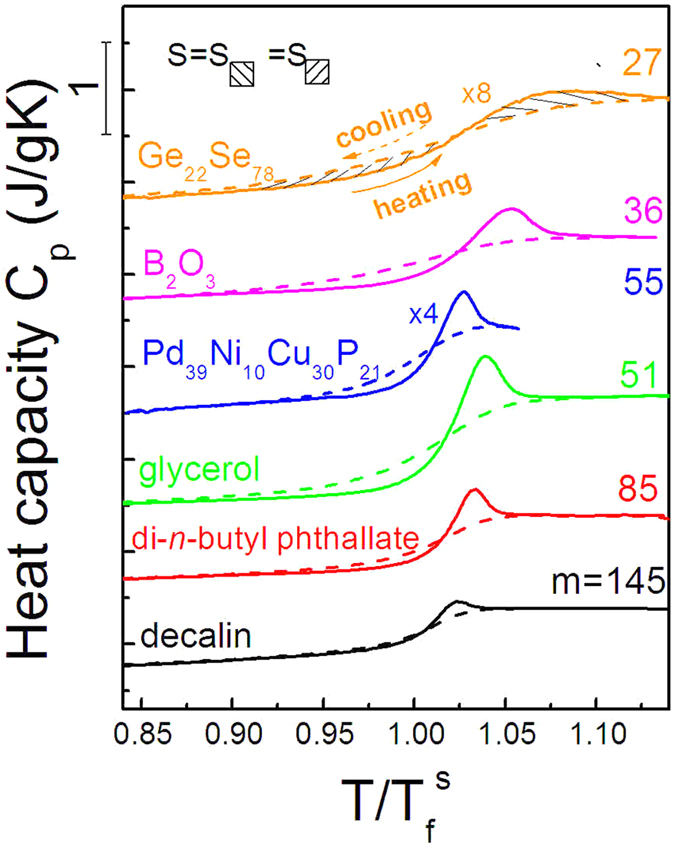
Heat capacity curves measured in the cooling and heating processes for Ge_*x*_Se_1−*x*_ and other glass-forming materials with diverse fragility, including small molecules, metals and oxides. All the curves were measured at the cooling/heating rates of −/+ 20 K/min. The materials are Ge_22_Se_78_, B_2_O_3_, Pd_39_Ni_10_Cu_30_P_21_, glycerol, di-*n*-butyl-phthallate and decalin.

**Figure 6:**
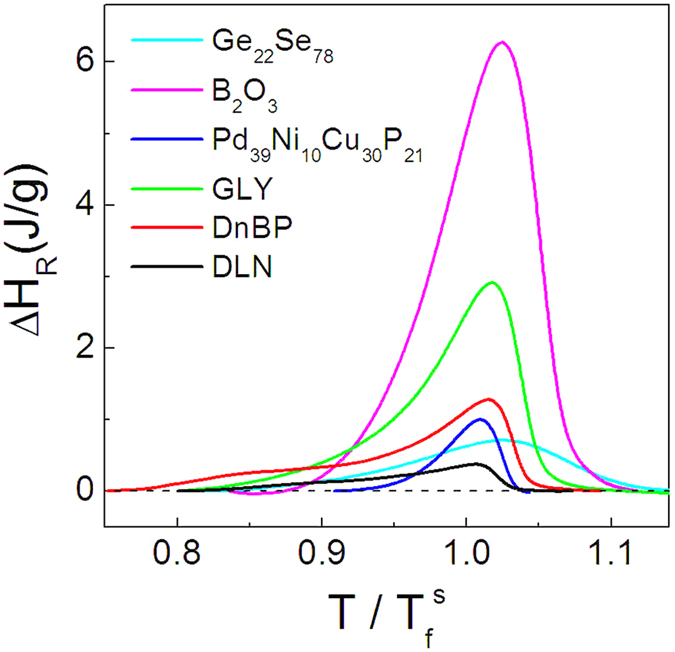
Enthalpy difference curves involved in the cooling and heating cycles for various glass formers around the glass transitions.

Figure 5 shows the heating and cooling *C*_*p*_ curves for a group of glass forming liquids of diverse fragility with temperature normalized to their 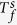 values. Five more glass forming systems are used covering inorganic oxide, molecular^37^ and metallic^38^ glasses. Accordingly, the enthalpy relaxation spectra are plotted in Fig. 6 using the temperature reduced by the fictive temperature and, consequently, the peak values in the spectra read 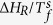.

**Figure 7:**
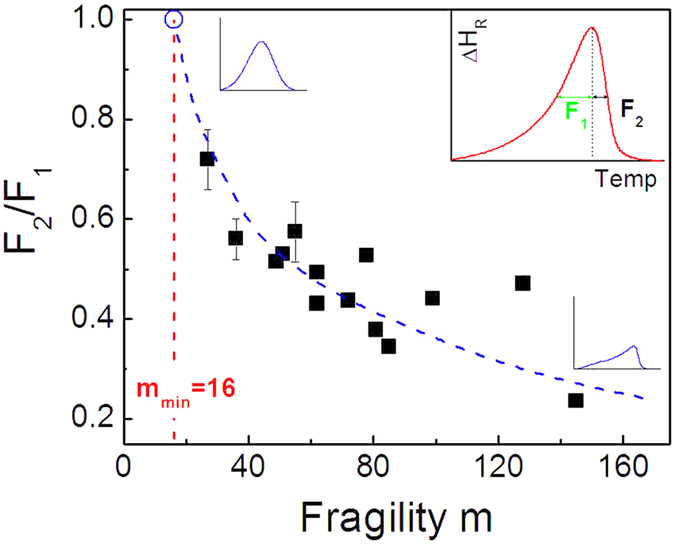
Dependence of the enthalpy difference on the fragility *m*-indexes for various glass formers. The dashed line is a guide for the eye. 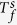 is the reference fictive temperature defined in the glass quenched at a cooling rate of 20 K/min.

To understand the enthalpy relaxation spectra in temperature domain defined by the enthalpy difference curves, we define a parameter of *S* = *F*_*2*_/*F*_*1*_ to reflect the symmetry of the spectra as shown in the inset of Fig. 7, where *F*_*1*_ and *F*_*2*_ denote the half width at half maximum of the peak in the left and right flanks, respectively, and therefore, *F*_*1*_ + *F*_*2*_ defines the full width at half maximum. Figure 7 presents the relation between the symmetry parameter *S* and fragility. The majority of the data are seen to fall into a master curve, and increased symmetry of the relaxation spectra is evident for strong liquids. Based on the correlation shown in Fig. 7, a complete symmetry might be expected for the strongest liquid of *m*_*min*_ = 16.

**Figure 8:**
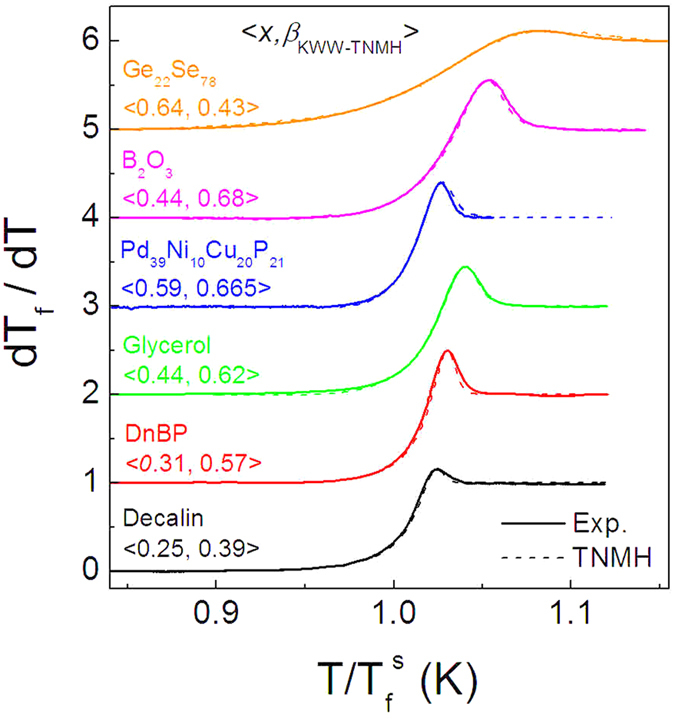
Normalized heat capacity derived in terms of the temperature derivative of the fictive temperature of various glass formers determined using the experimentally measured heating *C*_*p*_ curves of the glasses quenched at cooling rates of 20 K/min. The heating rates are 20 K/min for all the *C*_*p*_ measurements. The fits of the TNMH equations give the nonlinear factor *x* and the stretching exponent *β*_*KWW*_.

The heating (upscan) *C*_*p*_ curves of the glasses quenched at a cooling rate of 20 K/min are analyzed using the Tool–Narayanaswamy–Moynihan–Hodge (TNMH) equations, which emphasize the effects of fragility, nonlinear parameter *x* and stretching exponent *β*_*KWW*_^24^,^39^. The application of the TNMH equations requires the equality between the normalized *C*_*p*_ curves and the temperature derivative of the fictive temperature, *dT*_*f*_ /*dT* (detailed description is available in early studies^39^). The fit of the TNMH equations to the normalized *C*_*p*_ data therefore proceeds with the parameters of pre-exponential (*A*), apparent activation energy (*E*_*a*_ = *ln10RT*_*g*_*m*), *x* and *β*_*KWW*_. With *m* = 27 available for the Ge_22_Se_78_ glass, the fit gives the two parameters, *β*_*KWW*_ and *x*, to be 0.43 ± 0.05 and 0.64 ± 0.05 respectively as shown in Fig. 8. It is noted that the mediate glass transition temperature *T*_*g*_ and the marked glass-transition heat capacity increment for the Ge_22_Se_78_ glass are advantageous for the enhanced accuracy in the DSC measurements and, consequently guarantee the reliable results of the non-exponential and non-linear parameters involved in the enthalpy relaxation. It is a surprise to see such a low *β*_*KWW*_value for the Ge_22_Se_78_ glass presents the large deviation from the general correlation between *m* and *β*_*KWW*_^14^.

## Discussion

The determination of the fragility for the Ge_22_Se_78_ glass indicates that the glass former belongs to the category of strong dynamics according to the Angell’s strong-fragile classification scheme^12^. The fragility of various glass formers has been explored for decades and, experimentally, the accessible *m*-index spans from the hitherto strongest SiO_2_ of *m* = 20 to the most fragile cis/trans-decalin of *m* = 145^40^. The low fragility (*m* = 27) for the Ge_22_Se_78_ alloy is somehow unexpected since, for inorganic substances, the extremely low fragility is generally associated with the network glass-forming materials with the MX_2_ stoichiometry such as SiO_2_ and GeO_2_, where the structures are built predominantly by tetrahedral MX_4_ motifs^12^,^24^,^38^,^40^,^41^,^42^,^43^. Numerically, the Ge_22_Se_78_ fragility is quite comparable with those of BeF_2_ (*m* = 24) and ZnCl_2_ (*m* = 30) glasses^40^, which have strong directional bonds for the network structure^41^,^44^.

Studies found that strong glass formers with low *m* indexes generally have relatively high *β*_*KWW*_ values (typically higher than 0.6 for the liquids with *m* < 40)^14^,^45^. Therefore, at a glance, the low enthalpy-relaxation-based *β*_*KWW*_ of 0.43 is unusual when compared with the strong Ge_22_Se_78_ glass of *m* = 27. In the earlier studies of the *β*_*KWW*_ data produced by enthalpy relaxation and dynamic measurements such as dielectric and mechanical relaxations for various glass formers, excellent consistency is often exhibited^24^,^38^,^46^, suggesting the TNMH-based enthalpy relaxation can produce reliable stretching exponents. It appears that the low *β*_*KWW*_ value of 0.43 makes the Ge_22_Se_78_ glass to be an exception in the strong glass formers with simple compositions^14^,^45^.

Dynamic behaviors in glass formers have been recognized to be associated with the structural features of glasses and melts. Our recent studies of binary glass forming mixtures^47^,^48^ showed that mixing always generates a negative shift of the actual fragility relative to the linear averaging of the fragility values of the two pure components, independent of the sign of the enthalpy of mixing. And in some cases^48^, the minimum fragility can be achieved at an intermediate composition in mixtures. Similarly, the studies of binary glass formers with weak intermolecular interactions detected the lower *β*_*KWW*_ values than those of pure components, suggesting effective broadening of the relaxation dispersion^47^,^49^. In contrast, in the mixtures with strong intermolecular interactions manifested by large and negative enthalpies of mixing, enhanced *β*_*KWW*_ values are evident^47^. For the Ge_22_Se_78_ glass, it is, consequently, speculated that the low fragility *m*-index and the small *β*_*KWW*_ value might be partly associated with the mixing effect. This is quantitatively consistent with the random connectivity of the basic structure motifs of GeSe_4_ tetrahedra and (Se)_n_ units in the Ge_22_Se_78_ glass, as involved in the reported models^6^,^29^,^50^. Due to the unique structure of GeSe_2_ (much different from those of GeO_2_ or SiO_2_)^51^,^52^, complexes have been argued to develop based on the basic structural motifs in the Ge_22_Se_78_ glass^7^,^8^,^9^,^29^,^53^.

The present studies also detect a relation of *x* > *β*_*KWW*_ for the Ge_22_Se_78_ glass. This relation is quite rarely reported for most of glass formers, where *x* is generally smaller than *β*_*KWW*_^24^,^54^. As a few more glass formers are recently studied such as Zr_46.75_Ti_8.25_Cu_7.5_Ni_10_Be_27.5_^55^, Ge_15_Te_85_^56^,^57^, and Au_49_Cu_26.9_Ag_5.5_Pd_2.3_Si_16.3_^58^, similar relations are reached. Further study appears to be necessitated to clarify the unusual behavior.

## Methods

### Sample preparation

The sample glass Ge_22_Se_78_ was prepared with the melt-quenching method^27^. Pure elements of Ge and Se (5N purity, Alfa) mixed according to specific fractions with weight of ~1.5 g are sealed into quartz tubes of 8 mm in diameter under a vacuum of 10^−3^ Pa. The mixtures were heated up to 1230 K, higher than 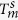 of two pure components, and kept isothermally for 24 hours in a rocking furnace to promote the degree of mixing. Subsequently, the temperature was set to 1020 K, which is about 100 K above the liquidus temperature for additional 10 hours (24 + 10 hours) and 168 hours (24 + 168 hours), respectively. Glasses were finally obtained by quenching the melt into ice water. The composition of the sample with the synthesized time for 24 + 168 hours was analyzed taken randomly from the bulk using the energy dispersive spectrometer (EDS) analyses (supplemental material).

### Sample analyses

The structure of the melt-quenched samples were checked via the x-ray power diffraction in a Rigaku D/MAX/2500/PC (Cu K_α_, λ = 1.54 Å) and Raman measurements on a Renishaw inVia micro Raman spectroscopy with a laser radiation of 514 nm. Heating and cooling heat capacity curves were obtained by using a differential scanning calorimeter (DSC, Perkin Elmer 8000) calibrated by using indium and zinc as references. The sample mass varied from 6 to 10 mg. The calorimetric glass transition temperature *T*_*g*_ was defined as the onset temperature of the heat capacity jump from the glassy to the liquid state. Enthalpy relaxation was performed in two manners. One used a cooling/heating cycle at the same rates of 20 K/min, and the enthalpy difference between the cooling and subsequent heating defines the relaxation enthalpy. The other is based on the identification of the enthalpy difference in the glasses quenched by various cooling rates, of which the enthalpy difference is calculated from the integral of the heating *C*_*p*_ difference among the glasses quenched at various cooling rates but fixed heating rate, typically 20 K/min. Usually, the heat capacity curve generated from cooling/heating rates of 20 K/min is set as the reference. This method has been described elsewhere^23^. The *C*_*p*_ measurement temperature spans from *T*_*g*_ − 100 K to *T*_*g*_ + 50 K, which can guarantee that no relaxation occurs at low temperature regimes, and equilibrium (supercooled liquids) is reached at high temperature regimes.

## Additional Information

**How to cite this article**: Li, P. *et al*. Relaxation dynamics in the strong chalcogenide glass-former of Ge_22_Se_78_. *Sci. Rep.* **7**, 40547; doi: 10.1038/srep40547 (2017).

**Publisher's note:** Springer Nature remains neutral with regard to jurisdictional claims in published maps and institutional affiliations.

## Supplementary Material

Supplementary Information
