# Supplementary material for: Relaxation dynamics in the strong chalcogenide glass-former of Ge22Se78
Source: Sci Rep. 2017 Jan 17;7:40547. doi: 10.1038/srep40547 (PMC5240093; doi:10.1038/srep40547)
Supplement: Supplementary Information [file srep40547-s1.doc]

Relaxation dynamics in the strong chalcogenide glass-former of Ge22Se78

Pengfei Li, Yaqi Zhang, Zeming Chen, Peng Gao, Tao Wu and Li-Min Wang*

State Key Lab of Metastable Materials Science and Technology, and College of Materials Science and Engineering, Yanshan University, Qinhuangdao, Hebei, 066004 China

Energy Dispersive Spectrometer (EDS) analyses


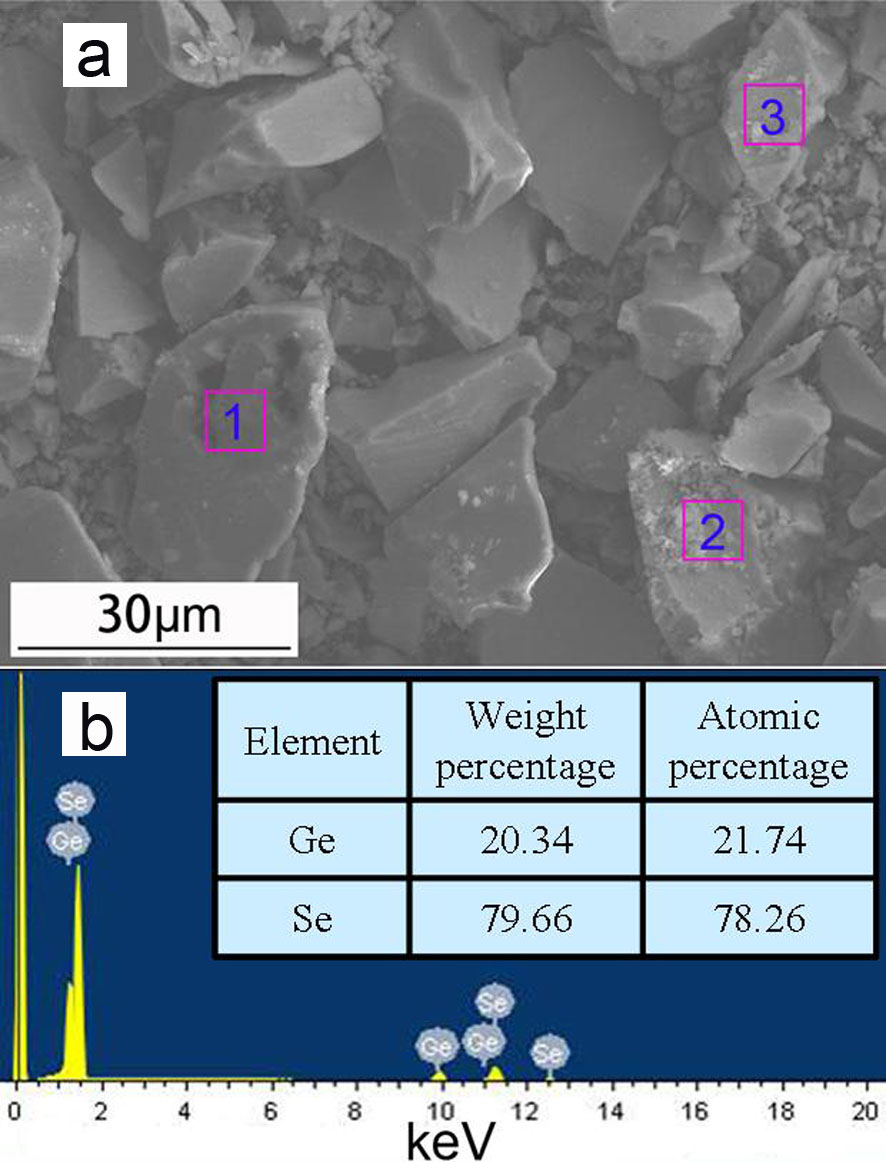


Figure S1

Fig S1. SEM image (a) and EDS spectra (b) of the powder samples for the glass with synthesis time of 24+168 hours. The inset table in (b) shows the composition in the area denoted by the number of 1 in (a). And the composition in the areas denoted by 2 and 3 are shown below. Note that the three areas are taken randomly from the bulk and ground into powders. The compositions in the three areas are very comparable with the nominal composition of Ge_22_Se_78_ when considering the measurement error, showing homogeneous composition in the glassy samples.

| Element | Weight percentage | Atomic percentage |
| --- | --- | --- |
| Ge | 20.54 | 21.94 |
| Se | 79.46 | 78.06 |

| Element | Weight percentage | Atomic percentage |
| --- | --- | --- |
| Ge | 20.69 | 22.10 |
| Se | 79.31 | 77.90 |

Table 2 Table 3
